# Rotor subunits adaptations in ATP synthases from photosynthetic organisms

**DOI:** 10.1042/BST20190936

**Published:** 2021-04-23

**Authors:** Anthony Cheuk, Thomas Meier

**Affiliations:** Department of Life Sciences, Imperial College London, Exhibition Road, London SW7 2AZ, U.K.

**Keywords:** bioenergetics, F1Fo-ATP synthase, ion-to-ATP ratio, photosynthesis, regulation, rotor *c*-ring

## Abstract

Driven by transmembrane electrochemical ion gradients, F-type ATP synthases are the primary source of the universal energy currency, adenosine triphosphate (ATP), throughout all domains of life. The ATP synthase found in the thylakoid membranes of photosynthetic organisms has some unique features not present in other bacterial or mitochondrial systems. Among these is a larger-than-average transmembrane rotor ring and a redox-regulated switch capable of inhibiting ATP hydrolysis activity in the dark by uniquely adapted rotor subunit modifications. Here, we review recent insights into the structure and mechanism of ATP synthases specifically involved in photosynthesis and explore the cellular physiological consequences of these adaptations at short and long time scales.

## Introduction

Cellular life depends on the energy that is ultimately powered by sun light. Its direct users are plants, algae and some clades of bacteria, e.g. cyanobacteria and purple bacteria. These photoautotrophic organisms convert solar energy into chemical forms of energy by photosynthesis, a process in which 7 × 10^16 ^g of carbon is fixed by autotrophs globally each year [1]. The light-dependent reactions of photosynthesis involve a series of large, macromolecular complexes, which are capable of harvesting light energy to split water molecules releasing electrons for the reduction in NADP^+^ into NADPH and releasing protons (H^+^) into the thylakoid lumen generating the proton-motive force, *pmf*, required for ATP synthesis. The resultant pools of NADPH and ATP are essential substrates for the Calvin–Benson–Bassham cycle (CBB) where carbon fixation occurs [2,3]. The four large protein complexes involved in the production of NADPH and ATP are photosystem II, cytochrome *b*_6_*f*, photosystem I and ATP synthase. The first three complexes form the electron transport chain for NADP^+^ reduction and the latter complex synthesising ATP, the cells’ universal energy currency [4–6]. This review focuses on ATP synthase from photosynthetic organisms, which possesses some unique features not observed in other forms of life.

**Figure 1.**
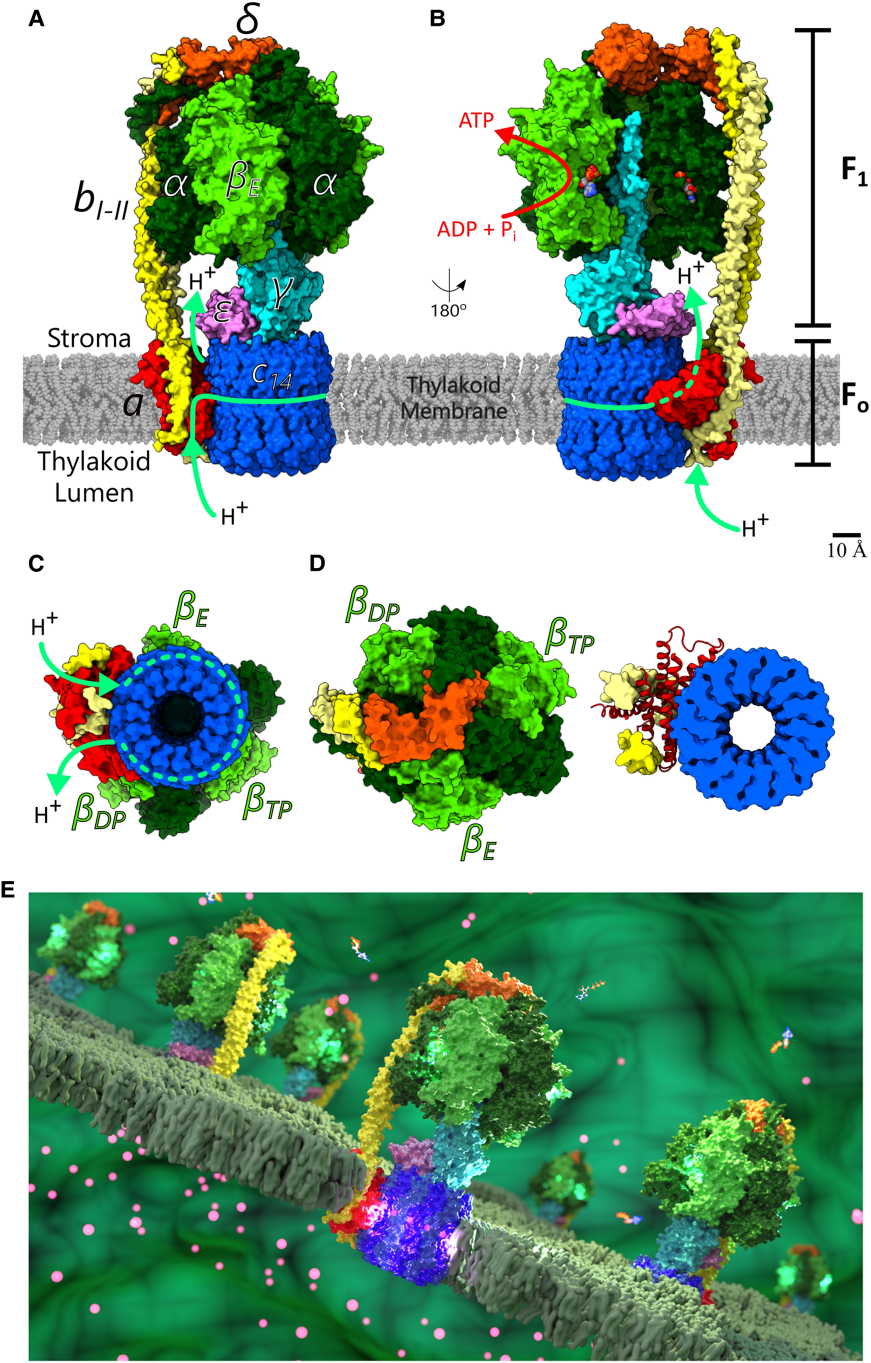
Structure of the spinach chloroplast F_1_F_o_-ATP synthase. (**A**) The F_1_F_o_-ATP synthase from spinach chloroplast is found embedded in the thylakoid membranes and couples the proton-motive force, *pmf*, with the synthesis of ATP. The water-soluble, catalytic F_1_ subcomplex consists of subunits *α_3_β_3_γδε*. The membrane-embedded F_o_ subcomplex consists of subunits *ab_2_c_14_*. During ATP synthesis, protons enter via an aqueous half-channel on the *a*-subunit exposed to the thylakoid lumen and bind successively to the binding sites on the *c_14_*-ring. As protons bind, the *c_14_*-ring rotates almost a full turn before the protons exit through a second half-channel formed by an aqueous wedge between the *c*-ring and the *a*-subunit into the stroma. The torque generated by the rotating *c_14_*-ring is transferred to the catalytic F_1_ head group via the central stalk (*γε*). In the catalytic *β*-subunits ADP and P_i_ condenses to ATP. Each of the three *β*-subunits is in different conformational state at any given time point, either empty (*β_E_*), bound to ADP (*β_DP_*) or bound to ATP (*β_TP_*) [15,76]. Arrows indicate the direction of proton flow through F_o_ during ATP synthesis. (**B**) Open view with *β_DP_* and one adjacent *α*-subunit removed; bound ADP and ATP nucleotides are indicated (sphere models). (**C**) Bottom view, from lumen side. (**D**) Top view from stroma, including cutaway showing *a*-subunit (cartoon model) in association with *c_14_*-ring at the level of the ion binding sites of the *c*-ring (see Figure 2). (**E**) Illustrative representation of ATP synthases embedded in a thylakoid membrane. The structure models are from [12], in surface representation.

The *pmf* is the direct energy source of the rotary F_1_F_o_-ATP synthase; a large, membrane-embedded protein complex that converts the substrates adenosine diphosphate (ADP) and inorganic phosphate (P_i_) into ATP by a unique rotational mechanism. The process of ATP synthesis by ATP synthases is entirely reversible. Under particular cellular conditions, the ATP synthase can operate in reverse, converting energy from ATP hydrolysis and pumping ions across the membrane to generate a *pmf* [7]. The *pmf* itself is composed of two components, a transmembrane electrical potential (Δ*ψ*) and a transmembrane chemical proton gradient (ΔpH). The ATP synthase from bacterial, chloroplast and mitochondrial sources plays a key role in cellular bioenergetics and has been studied by biochemical and structural methods already for many decades [8–11]. More recently, electron *cryo*-microscopy (*cryo*-EM) has revealed the structure of fully intact F-type ATP synthases from chloroplasts at high-resolution, resolving the intermediary conformational states and other key mechanistic and regulatory aspects (Figure 1) [12,13].

## Structure and function of ATP synthase and its F_1_ complex

The F-type ATP synthase found in bacteria and chloroplasts is ∼550 kDa in size with a protein subunit stoichiometry of *α_3_β_3_γδεab_2_c_8–17_* (Figure 1). The complex can be divided into two biochemically and functionally distinct subcomplexes: The water-soluble, catalytic head subcomplex, F_1_, composed of *α_3_β_3_γδε* subunits and the membrane-embedded rotor–stator subcomplex, F_o_, composed of *ab_2_c_8–17_* subunits. F_1_ and F_o_ are connected by a central and peripheral stalk. From an operational point of view, terms such as rotor and stator are applicable, with the rotor (*c_8–17_γε*) and the stator (*α_3_β_3_δab_2_*) subcomplexes.

In F_1_, the synthesis and hydrolysis of ATP occurs at a hexamer of alternating *α*- and *β-*subunits (*α_3_β_3_*) where one ATP molecule is able to bind at a Walker A motif (GxxxxGK[TS]), also known as the P-loop, found in each of the three *β*-subunits [14]. Additionally, each of the three *α*-subunits contains a non-catalytic nucleotide-binding site that usually bind unreactive (‘structural’) bound ATP molecules (Figure 1B) [15]. The central stalk, composed of the *γ*-subunit and *ε*-subunit, induces the necessary conformational changes in the *α_3_β_3_* hexamer head for ATP synthesis by transferring torque generated in the membrane-embedded F_o_ subcomplex into the F_1_ catalytic head.

The peripheral/outer stalk, consisting of *δ*-, *b*- and *b′*-subunits, acts as a stabilising stator element between F_1_ and F_o_ preventing futile co-rotation. The *δ*-subunit has an N-terminal domain that connects to the three *α*-subunits in F_1_, and a C-terminal domain that binds to the *b*-subunits of the peripheral stalk. A flexible hinge connects the two domains of the *δ*-subunit and gives rise to a highly dynamic coupling of F_1_ and F_o_ [16]. The two *b*-subunits are copies of each other with high sequence identity; structurally they split at their membrane sections and sit on the *a*-subunit, similar to a horse saddle. In the soluble, outer stalk region they connect to form a right-handed coiled-coil motif (Figure 1B) with the purpose to counteract torque that is transmitted into F_1_ during ATP synthesis [17]. At the other end, the two *b*-subunits bind at the F_1_ hexamer head and form several contacts to different domains at the outer surface of α-subunit as well as contacts to the *δ*-subunit on top of the F_1_ headpiece at several locations.

F-type ATP synthases have only one outer stalk, while other rotary ATPases (A-type, V-type) have two or three [11]. One can, therefore, speculate safely that the determination of the peripheral stalk stoichiometry in F-type ATP synthases must lie in the structural attachment of the *δ*-subunit, which is asymmetrically bound on top of the F_1_ headpiece, which once bound, irreversibly determines the position of two *b*-subunits composing the peripheral stalk (Figure 1D).

## The F_o_ complex: ion translocation pathways and the role of the *c*-ring

The F_o_ subcomplex accommodates the membranous ion motor of the ATP synthase. The motor comprises the rotor ring, also known as the *c*-ring due to its oligomeric assembly of a species-specific number of *c*-subunit copies [18], and the stator *a*-subunit that lies outside, adjacent to the *c*-ring and forms two water accessible, separated aqueous half channels (Figure 1) [19]. The two half channels are laterally adjacent but spatially and electrostatically separated by a highly conserved and positively charged arginine side chain, and each channel provides an ion access pathway to and from the *c*-ring ion binding sites to opposite sides of the membrane [20–22].

**Figure 2.**
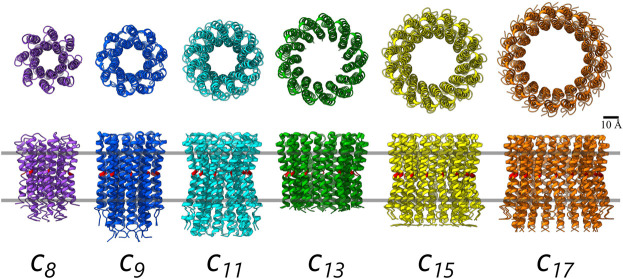
Examples of F-type ATP synthase *c_n_*-ring stoichiometries from *c_8_* to *c_17_*. The *c*-rings are shown in cartoon representation. Upper row: View from the cytoplasm (matrix/stroma). Lower row: view from the membrane plane in the same orientation as in Figure 1A. The membrane borders are indicated by grey lines. From left to right: *c_8_*-ring from *B. taurus* mitochondria [35], *c_11_*-ring from *M. phlei* [50], *c_11_*-ring from *Ilyobacter tartaricus* [18], *c_13_*-ring from *Bacillus pseudofirmus* OF4 [43], *c_15_*-ring from *Spirulina platensis* [25], *c_17_*-ring from *Burkholderia pseudomallei* [36]. The location of the conserved carboxylate amino acid residue required for ion binding is coloured red. All the examples here are proton-binding *c_n_*-rings, except for the *c_11_*-ring from *I. tartaricus* which is Na^+^-binding. Plant chloroplast ATP synthase harbours a *c_14_*-ring (Figure 1D), while cyanobacterial ATP synthases have *c_13_*–*c_15_* rings [49]. The resulting overall range of *ion-to-ATP ratios* in fully coupled ATP synthases is 2.7–5.7. For a functional description, see text.

The *c*-rings are structurally highly conserved and resemble hourglass-shaped cylinders with a central pore (Figure 2). The ion binding sites on the *c*-ring lie at their outside surface, each in between two adjacent *c*-subunits; all binding sites are accessible from the outside. Ion coordination is mediated by a particular set of conserved amino acid residues, which also determine ion specificity either for protons or Na^+^ [23–25]. Common to all binding sites in all rotary ATPases is a highly conserved carboxylate group, which plays the key role in H^+^ (and sometimes Na^+^) coordination. While in the ion bound state the site remains in a *locked* conformational state [18,25], it has been shown that the binding and unbinding events themselves are dependent on hydration events at the ion binding site [26]. These events can also be viewed from the perspective of shifts in p*K*_a_ values of the binding site carboxylate groups, with a p*K*_a_ of a hydrated carboxylate being ∼4.5 and a much-increased p*K*_a_ (7.5 or higher, [24,27] for the same carboxylate in a hydrophobic environment, e.g. within a membrane or a detergent micelle. Hence, while the ion remains locked at its *c*-ring ion binding site during its travel along the hydrophobic membrane interface, upon reaching the hydrophilic amino acid residues of the exit channel, the site encounters a new chemical environment that lowers the p*K*_a_ and leads the carboxylate to *open* its conformation and ultimately release the ion into the channel towards the opposite side of the membrane [26,28]. Recent structural studies of ATPases have elucidated a range of these ion translocation pathways [12,16,29–32]. While the overall mechanism of ion access and release involving the *a*-subunit and the ion binding mode on the *c*-ring is highly conserved in all rotary ATPases, including the V-type and A-type rotary ATPases/synthases, the details of their individual channels and the exact ion paths along conserved hydrophilic or charged amino acid residues varies between different species [10].

## The *c*-ring stoichiometry and the ATP synthase's *ion-to-ATP ratio*

The *c*-ring in F_o_ is composed of a variable number (*n*) of monomeric *c*-subunits, with each monomer forming an ∼8–9 kDa hairpin of two transmembrane *α*-helices, connected by a loop that non-covalently connects to the F_1_ subcomplex via *γ*- and *ε*-subunits [33,34]. The known range covers most integers between *n* = 8 (*Bos taurus*) [35] and *n* = 17 (*Burkholderia pseudomallei*) [36] (exception: *n* = 16). The available evidence suggests that the stoichiometry of *c_n_*-rings is determined solely by the primary structure of the *c*-subunit and thus remains constant within a given species but varies across different species [10,18,25,37–39]. This notion is not only supported by the formation of functional chimeric ATP synthases, where resulting chimeric ATP synthases have a *c_n_*-ring stoichiometry matching the source organism [40–43], but it is also strongly suggested by heterologous expression of a variety of *c*-rings in *Escherichia coli* cells, all showing native (i.e. non *E. coli*) *c*-ring stoichiometries [44–46].

In tightly coupled F_1_ and F_o_ complexes a key bioenergetic parameter known as the *ion-to-ATP ratio* can be derived, which equals the number of *c*-subunits in the *c_n_*-ring (*n*) divided by the number of ATP molecules synthesised per full turnover, three. The ratio, therefore, determines the bioenergetic cost of making a single ATP molecule for a given organism [35] and it also contributes strongly to the thermodynamic efficiency of membrane systems [47]. Remarkably, when experimentally varying the *c*-subunit stoichiometry of a given ATP synthase by site-directed mutagenesis of the *c*-subunit and thereby experimentally inducing variation of the *ion-to-ATP ratio*, it effects both on the enzymatic level of ATP synthase kinetics [42] as well as on the growth level of the given bacterium [43]. This fact holds high hopes for similar future engineering approaches of ATP synthases to be adapted to externally imposed bioenergetic challenges, such as low light conditions in cyanobacteria or plants.

In the plant ATP synthase, the *c*-ring stoichiometry is *c_14_* [48], while in cyanobacteria the stoichiometry varies between 13 and 15 [49]. Compared with most bacterial or mitochondrial ATP synthases this is a rather large stoichiometry, leading to an *ion-to-ATP ratio* of 4.7 in plants and 4.3–5.0 in cyanobacteria. With the total range of known *c*-rings known (*c_8_*–*c_17_*, Figure 2) the variation of this ratio becomes impressively wide, 2.7–5.7 ions per ATP. The photosynthetic active organisms take their place in the upper regions of this range.

## Bioenergetic consequences of *c*-ring stoichiometry variation

The differently primed functions as well as the cellular physiological environments of ATP synthase in various organisms may explain the large variation in *c_n_*-ring stoichiometries and hence also help understand the variations found in the *ion-to-ATP ratios* in plant and cyanobacterial ATP synthases. For an ATP synthase operating under sufficiently high *pmf* (or *smf*), smaller *c_n_*-ring stoichiometries are advantageous as they ‘consume’ fewer, albeit higher energised, protons per ATP synthesised. The hypothesis is supported by the *smallest* known stoichiometries, which belong to F-type ATP synthases found in animal (and plant) mitochondria: Mammalian mitochondrial ATP synthase operates with a *c_8_*-ring [35] and is known to operate almost exclusively in ATP synthesis direction. Some bacteria, such as *Mycobacterium phlei*, *E. coli* or some *Bacillus* species have *c_n_*-ring stoichiometries of *n* = 9, 10, 11, 12 and 13 [39,50-53]. Some of these bacteria are living in physiologically challenging environments, e.g. high temperature and high pH and hence specifically adapted in stoichiometry and ion binding chemistry [39,50,54], while others are challenged by aerobic–anaerobic environmental changes [55-57]. Hence in these latter cases, occasionally acting as ion pumps (ATPases) to maintain the *pmf* is more efficient with larger *c_n_*-ring stoichiometries as more ions can be translocated per ATP hydrolysed. In its extreme with the *largest c*-ring found so far (*c_17_*-ring, *B. pseudomallei*) the ATP synthase is suggested to exclusively consume ATP to pump protons out, allowing the pathogen to survive in acidic environments of macrophages during the early stages of an infection [36].

In photosynthetic organisms with their larger *c_n_*-ring stoichiometries, this disparity in stoichiometries and their relatively high *ion-to-ATP ratios* reflects the requirement for ATP synthesis to occur even under conditions where only a low total *pmf* is available. In photosynthetic organisms or organelles (Table 1) light fluctuations can lead to rapid changes of the *pmf*. Under high-light conditions, photosynthetically active organisms can establish a remarkably high ΔpH across the thylakoid membrane, up to a pH gradient of 2.5 [58,59]. This corresponds to −180 mV of total *pmf*, mainly provided by the ΔpH component of −150 mV. While the electric component (Δ*Ψ*) in this proton gradient is much lower (−30 mV), the high concentration of protons reflected by the high ΔpH component in the lumen of thylakoid membranes compensates for the overall phosphorylation potential (Δ*G*^0′^) required for the synthesis of ATP between 42 kJ/mol (low light) and 60 kJ/mol (high light) that has been determined under various experimental conditions [60]. The *c*-rings in the ATP synthases from these organisms are generally enlarged in their stoichiometry, transporting more protons per ATP (higher *ion-to-ATP ratio*). In contrast, the *pmf* in animal mitochondria is mostly stored as Δ*ψ* (∼150 mV) and a steady supply of protons favours a small *c_n_*-ring stoichiometry as this requires fewer protons per ATP molecule synthesised (Table 1). In the case of bovine mitochondrial F-type ATP synthase, which has a *c_8_*-ring, the *ion-to-ATP ratio* is 8/3 = 2.7 [35]. A similarly high contribution to *pmf* by Δ*ψ* is not favourable in photosynthetic systems as high membrane potentials lead to more recombination events and results in photodamage [61]. The differences in *c_n_*-ring stoichiometries are an example of how ATP synthases have adapted to different physiological and environmental conditions over longer, evolutionary time scales. In photosynthetic organisms, the unique bioenergetic demands and environmental pressures have favoured larger *c_n_*-ring stoichiometries, resulting in the higher *ion-to-ATP ratios*.

**Table 1.** Comparison of overall *pmf*, the *pmf* components (ΔΨ and ΔpH) and the c-ring stoichiometry found in organisms from different kingdoms of life. The photosynthetic active organisms are shown in bold.

Kingdom (Phylum)	Species (organelle or adaptation to pH)	Overall *pmf* (mV)	ΔΨ (mV)	ΔpH (mV)	c-ring stoichiometry
**Plantae**	***Spinacia oleraceae*** **(chloroplast)**	**−180 mV** [58]	**−30 mV** [59]	**−150 mV**	**14** [48]
**Bacteria (Cyanobacteria)**	Synechococcus PCC 7942* **(neutrophile)**	**−190 mV** [77]	**−90 mV** [77]	**−100 mV** [77]	**13-15** [49]
Animalia	*Bos taurus* (mitochondria)	−210 mV [78]	−150 mV	−60 mV	8 [35]
Bacteria (Proteobacteria)	*E. coli* (neutrophile)	−170 mV	−135 mV	−35 mV	10 [51]
Bacteria (Firmicutes)	*Bacillus pseudofirmus* OF4 (alkaliphile)	−50 mV	−180 mV	+130 mV	13 [39]

*previously known as: Anacystis nidulans *or* Synechococcus *R-2.*

## Regulation of ATP synthases in photosynthetic organisms

A second unique feature of ATP synthases from plant chloroplasts and other photoautotrophic organisms is how they adapt their ATP synthesis activity in response to light intensity through a redox-modulated thioredoxin system [62–64]. Under sufficient light intensity, linear electron flow reduces thioredoxin which in turn reduces a redox switch found in the *γ*-subunit of ATP synthase. In this reduced state, ATP synthase remains active —– this can be artificially induced with the addition of the reducing agent dithiothreitol (DTT) [65]. On the other hand, with insufficient light intensity, the redox switch in ATP synthase is oxidised and enzyme activity is inhibited. This mechanism helps to down-regulate ATP synthase operation in the reverse direction (ATPase) and avoids wasteful ATP hydrolysis in the dark [57,63,64,66,67]. In plants, ATP is readily available for anabolic reactions of the CBB cycle.

**Figure 3.**
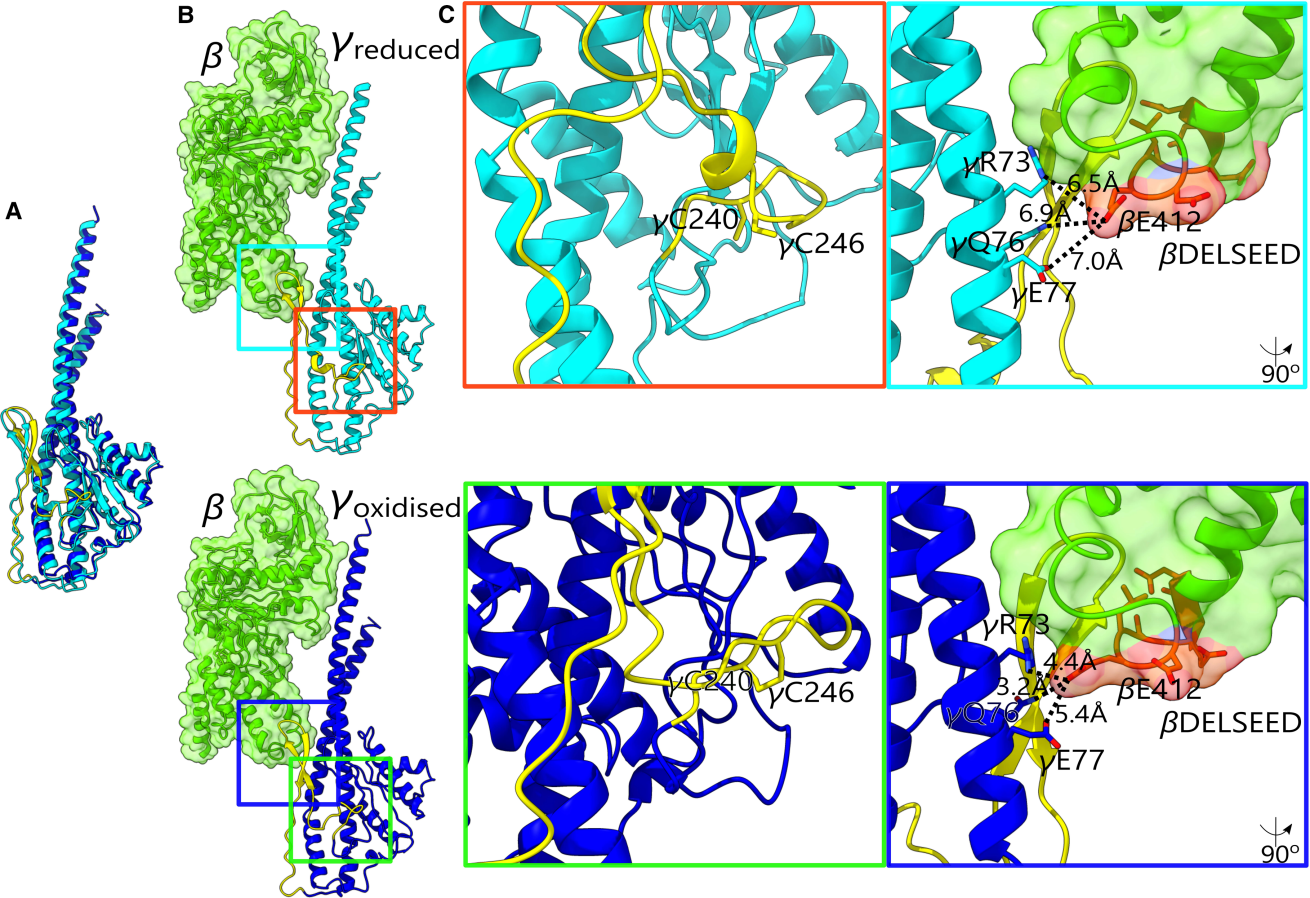
Chloroplast ATP synthase redox regulation by the *γ*-subunit. (**A**) Overlay of the reduced and oxidised conformations in cyan and blue, respectively. (**B**) The 40-residue insert forming an L-shape with two *β*-hairpins is shown in yellow and the interacting *β*-subunit is in green. (**C**) In the dark, the *γ*Cys^240^ and *γ*Cys^246^, on the shorter β-hairpin, are oxidised (*green* box) and form a disulfide bond which causes conformational changes in the longer β-hairpin of *γ*-subunit that lead to clashes with the *β-*DELSEED motif when rotating in the ATP hydrolysis direction (*cyan* square). While in the light, *γ*Cys^240^ and *γ*Cys^246^ are reduced (*red* square) and the *γ*-subunit does not clash with the *β-*DELSEED motif (*blue* square). Figure 3 is based on knowledge of both oxidised and reduced structures of the plant chloroplast ATP synthase [12,13].

To understand this regulatory feature from a structural point of view, the plant chloroplast F_1_F_o_-ATP synthase has been recently solved by *cryo*-EM at high-resolution in both an oxidised (inactive) state [12] and a reduced (active) state [13]. The latter was obtained by reducing the sample with DTT and stabilising this state with the uncompetitive inhibitor, tentoxin. The structures reveal that the rotor *γ*-subunit is the key structural element in the redox regulation in this enzyme (Figure 3).

The *γ*-subunit across all organisms typically consists of an antiparallel coiled-coil of the N- and C-terminal α-helices and a globular Rossmann-fold domain [68]. Relative to ATP synthases from non-photosynthetic bacteria and mitochondria, the *γ*-subunit from chloroplasts and cyanobacteria contains a unique, 40-residue insertion that forms an L-shape with two *β*-hairpins. The longer C-terminal *β*-hairpin extends towards the central cavity of the F_1_ hexamer where it can interact with a highly conserved DELSEED motif found near the bottom of the *β*-subunit (Figure 3C). Exclusively in plants, the shorter, N-terminal *γ*-subunit *β*-hairpin contains a motif (**C**DxNGx**C**) with cysteine residues on each of the two strands, capable of forming a disulfide bond in response to the chloroplast redox potential [64,69]. In the darkness, the disulfide bond forms under the oxidising conditions leading to a conformational change in the C-terminal *β*-hairpin that inhibits the rotation of the rotor in ATP hydrolysis direction by causing clashes between the coiled-coil of *γ*-subunit and the *β*-DELSEED. In light, the disulfide bond is once again reduced and there are no such clashes preventing rotary catalysis.

In contrast with chloroplast ATP synthase, the insertion found in cyanobacterial *γ*-subunits is only ∼25 amino acid residues and missing the shorter N-terminal *β*-hairpin and cysteine motif. However, experimental evidence suggests that the shorter insert in cyanobacterial *γ*-subunit provide regulatory capabilities through the induction of ADP-dependent inhibition [70]. Additionally, the rotor *ε*-subunit that is attached to the *γ*-subunit and the *c*-ring (Figure 1A/B) is observed in a retracted conformation that is not consistent with the *ε*-inhibition seen elsewhere in non-photosynthetic bacteria [29,67,71-73], where regulation involves the C-terminal part of the *ε*-subunit to reversibly interact with *β*-DELSEED in a so-called up-state (inhibited) or down-state (active) conformation [67,73,74]. In cyanobacteria though, the *ε*-subunit may be responsible for inducing the required conformational change in the *γ*-subunit to inhibit rotation in the ATP hydrolysis direction [72,75]. Structural studies of the cyanobacterial ATP synthase will help to identify which regulatory mechanisms play the most significant roles in cyanobacteria.

## Conclusions

Rotary F-type ATP synthases from photosynthetic active organisms such as plants (chloroplasts) and cyanobacteria show a set of unique rotor subunit adaptations for the regulation of their activities and functionality. On a short and immediate time scale, they control wasteful ATP hydrolysis during the anabolic biochemical reactions at day/night times by the *γ*-subunit. On the longer, evolutionary time scale, they adapted their *c*-ring stoichiometry to the ATP synthesis’ driving force, the *pmf*. The effects of these adaptations affect the operation modes of these ATP synthases and manifest in measurable cellular effects. Most conserved biochemical reactions in living organisms are achieved by enzymes with remarkable differences in their individual subunit compositions and structural features. In the case of ATP synthases from photosynthetic organisms, the adaptations to individual physiological requirements of the species are restricted to specific subunit modifications in their rotor subunits *c* and *γ*. These adaptations reflect the power of life to cope with environmental challenges at short and long time scales, but also bear interesting potential for future biotechnological advances.

## Perspectives

- F-type ATP synthases play a major role in the light-dependent reactions of oxygenic photosynthesis and have unique features compared with those found in mitochondria and non-photosynthetic bacteria.
- A wide range of ATP synthase structures are available, with mitochondrial and bacterial ATP synthases being particularly interesting with regards to health and medicine. Whilst, studying photosynthetic ATP synthases may help to reveal the complex interplay of the many cellular reactions of photosynthesis and their evolutionary past.
- Photosynthetic ATP synthases themselves may be a suitable target for engineering photosynthetic efficiency in plants or as a way to address an altered demand in ATP/NADPH, e.g. in C4 photosynthesis.

## Open Access

Open access for this article was enabled by the participation of Imperial College London in an all-inclusive *Read & Publish* pilot with Portland Press and the Biochemical Society under a transformative agreement with JISC.

## Abbreviations

ADP: adenosine diphosphate
AMP: adenosine monophosphate
ATP: adenosine triphosphate
NADP^+^/NADPH: nicotinamide adenine dinucleotide phosphate (oxidised/reduced)
OXPHOS: oxidative phosphorylation
